# IRF7 and CTSS are pivotal for cutaneous wound healing and may serve as therapeutic targets

**DOI:** 10.1038/s41392-023-01517-1

**Published:** 2023-08-30

**Authors:** Jiali Yin, Dongxin Shi, Yan Sun, Peiyao Zhu, Yiping Zhao, Xuegang Xu, Hongduo Chen, Yan Wu, Zhengwei Yuan, Xing-Hua Gao

**Affiliations:** 1 Department of Dermatology, The First Hospital of China Medical University, Shenyang, China; 2 Key Laboratory of Immunodermatology, Ministry of Education and NHC; National joint Engineering Research Center for Theranostics of Immunological Skin Diseases, Shenyang, China; 3 Department of Otolaryngology, Head and Neck Surgery, Yantai Yuhuangding Hospital, Qingdao University, Yantai, China; 4 Key Laboratory of Health Ministry for Congenital Malformation, Shengjing Hospital, China Medical University, Shenyang, China; 5 College of Medicine and Biological Information Engineering, Northeastern University, NO. 3-11, Wenhua Road, Heping District, Shenyang, China

**Keywords:** Disease model, Self-renewal, Genome, Trauma, Experimental models of disease

**Dear Editor**,

Adult cutaneous wounds typically result in deficient regeneration due to abnormal fibro-proliferative responses.^1^ The scarring, while conducive to preventing extended infection and loss of nutrients at the injury site, can be troublesome.^2^ In contrast, wound healing in embryos showes lower levels of inflammation and a remarkable capacity for remodeling.^3^ Interestingly, the capability of fetal scarless healing is age-dependent. As contrasted with the near-normal healing after the injury that occurs in the early gestational period (embryonic day 16, E16), wound healing in late gestation (embryonic day 18, E18) has prominent fibrosis. The mechanisms responsible for this transition are largely unknown.

From our gross and histological observations, the E18 age represents a critical time point for the transition from scarless to healing with scar. 48 h following a full-thickness skin excision surgery (Supplementary Fig. S1a–i), wounds of embryonic mice at the E16 healed without irregularity, while embryonic mice at the E18 displayed disparate changes, identified by India ink staining (Fig. 1a). The E18 showed dense collagen remodeling and a prominent accumulation of inflammatory cells (Supplementary Fig. S1j), which were evidenced by Sirius red staining-coupled laser confocal scanning microscopy and quantified by Fractal dimension (FD) and *lacunarity* (L) analyses. As expected, collagen bundles in E18 wounds at 48 h post-injury displayed a scar-like architecture with higher FD and lower L values as compared with those in the E16 wounds (Fig. 1b, Supplementary Fig. S1k).

**Fig. 1:**
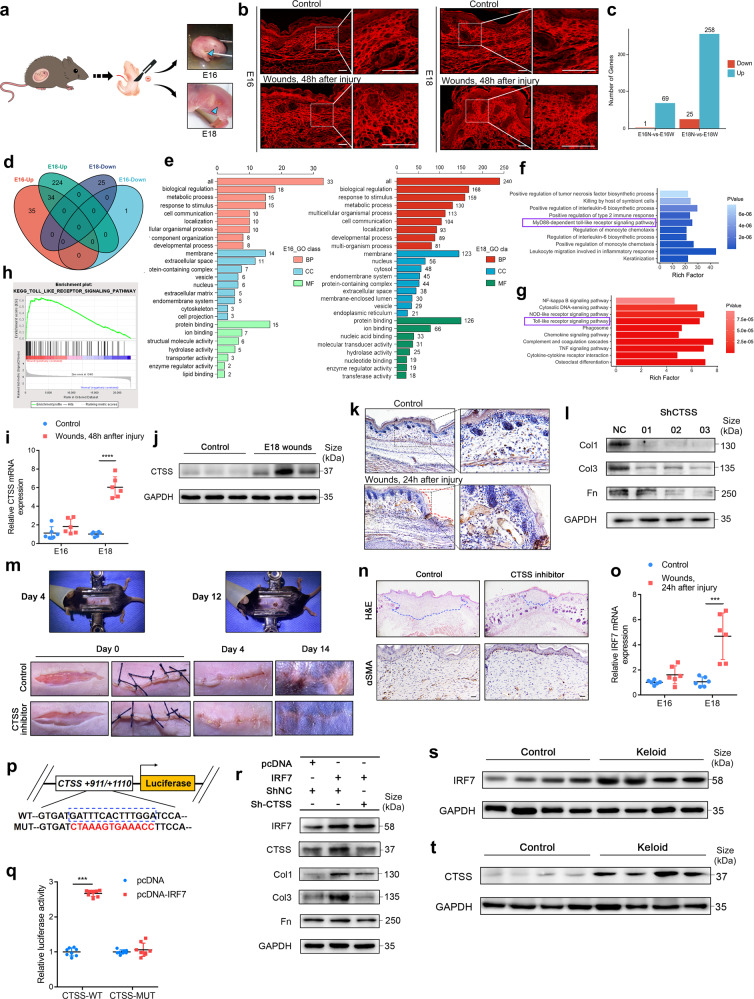
**a** Schematic diagram of surgical procedure and gross examination of the excisional wounds at 48 h post-injury (blue arrow indicates wound location as marked by Indian ink). **b** Sirius red staining (PSR) of wounds at 48 h post-injury and control skin sections as imaged using confocal laser microscopy. At 48 h post-injury, collagen arrangement within the E16 group was consistent with that of normal skin, while that in the E18 group was significantly disordered and dense. **c** DEGs identified in N (Controls) and E16 and E18 W (Wounded) groups (*n* = 3, per group). FDR ≤ 0.05 and fold-change ≥2. **d** Venn diagram of up- or down-regulated DEGs in the E16 and E18 groups. **e** Bar graphs of DEGs in the E16 and E18 groups as based on Go analysis of BP, CC and MF categories. **f**, **g** Top 10 enriched BP terms in GO analysis and pathways in the KEGG analysis of the E18 groups. **h** GSEA analysis of the entire gene-expression set and TLR signaling pathway in the E18 relative to the control group. FDR < 0.25 and *q*-value < 0.05. **i** Relative CTSS mRNA expressions in full-thickness skin tissues of controls and the E16 and E18 groups as assessed by RT-qPCR (*n* = 6). **j** Western blot of CTSS in skin tissue of E18 control and wound groups. **k** Representative immunohistochemical staining of CTSS protein in the E18 control and wound group. The red dotted line represents the defective skin tissue. **l** Western blot analysis of Collagen I (Col1), Collagen III (Col3) and Fibronectin (Fn) protein levels in HSFs cells transfected with ShRNA or Control. **m** Images of the control mice with the clip application (up). Images of wounds in control and LY3000328-treated mice from day 0 to day 14 (below). **n** H&E staining and α-SMA IHC staining images of E16 and E18 groups. **o** Relative IRF7 mRNA expression in full-thickness skin tissue of controls and the E16 and E18 groups (*n* = 6). **p** Schematic representation of CTSS mRNA depicting IRF7-binding sites in its promoter. **q** Luciferase reporter assays as performed in HSFs cells (*n* = 8). **r** Western blot analysis of IRF7, CTSS, Col1, Col3 and Fn protein levels in HSFs cells treated with different combinations of pcDNA, IRF7, ShRNA (CTSS). **s**, **t** Western blot of IRF7 and CTSS in normal human skin and keloid tissue. Data represent mean ± SD. Scale bars: 20 um. *P < 0.05, **P < 0.01, ***P < 0.001, ****P < 0.0001

By RNA-sequencing, we identified 70 and 283 differentially expressed genes (DEGs) within the E16 and E18 wounds, respectively (Fig. 1c), obtained at 24 h after the wounding as compared with that of self-control skin samples (*n* = 3 each). Following normalization with its control, 34 genes were co-upregulated in E16 and E18, while 224 were up-regulated and 25 down-regulated separately in the E18 wound (Fig. 1d). Such quantitative differences demonstrate the complexity of wound healing regulation that occurs at the transition from the E16 to E18 stage. We found that categories associated with wound healing were significantly increased in both the E16 and E18 groups, including responses to stimulation, metabolic processes, and cellular communication. Interestingly, the cellular component (CC) and molecular function (MF) in the E18 group were more represented in the nucleus and nucleic acid binding than that observed in the E16 group, suggesting an active involvement in nuclear transcription (Fig. 1e).

Similar enrichments were found in both groups concerning inflammatory cell-driven and keratinization pathways, as obtained by using GO enrichment and KEGG analysis to identify the top ten biological processes (BP) and pathways for E16 (Supplementary Fig. S2a, b) and E18 (Fig. 1f, g). However, a more prominent accumulation of interleukin 6, NF-kappa B pathway, and osteoclast differentiation was present in the E18 group, suggesting their potential roles for the transition from inflammatory responses to late repair after injury, as well as from a scarless to a fibrotic and scarring response. Notably, the Toll-like receptor (TLR) signaling pathway was identified in the E18 group, as revealed by both analyses. In the E18 group,102 genes in the TLR signaling pathway had 13 genes that intersected with DEGs within the Venn diagram (Supplementary Fig. S2c). Pathway analysis was also performed using genomic enrichment analysis (GSEA) and demonstrated that the TLR signaling pathway was globally activated and upregulated in the E18 wounds (Fig. 1h); validation for these genes in the TLR pathway reached the same conclusion (Supplementary Fig. S2d). Thus, it appears that a consistent, positive connection exists between the activation of the TLR signaling pathway and scar generation in the late embryonic developmental period.

Cathepsin S (CTSS) is a secreted protease involved in the TLR signaling pathway that can reshape a variety of extracellular matrix components, such as collagen and fibronectin in fibrotic diseases of multiple organs.^4^ By RNA sequencing, the differential expression of CTSS was prominent in E18 group (Supplementary Table 2), while not in the E16 group (Supplementary Table 3), the results of which were confirmed by RT-qPCR and Western Blotting, respectively (Fig. 1i, j, Supplementary Fig. S3a). Furthermore, IHC staining revealed that at 24 h post-injury, CTSS expression in the E18 group was significantly increased along the edge and residual site of the excision where clear positive staining of cells (fibroblasts and inflammatory cells) and intercellular stroma in the dermal region were identified (Fig. 1k, Supplementary Fig. S3b), while that in the E16 wound remained unchanged at either RNA or protein levels as compared with its own control (Supplementary Fig. S3c–f).

We next examined the effects of CTSS on human skin fibroblast cells (HSFs). With the downregulation of CTSS by transfection of knockdown plasmid, the expression of collagen I, collagen III, and fibronectin (Fig. 1l, Supplementary Fig. S4a–f), were all down-regulated. We administered a CTSS inhibitor, LY3000328, to hypertrophic scar C57B1/6 J model mice during the granulation and proliferation stages of scarring. Our results showed that continuous LY3000328 administration for 10 days decreased the scar growth remarkably (Fig. 1m), with an inhibition rate of 65.1%, also with the reduced tissue thickness (Supplementary Fig. S4g, Fig. S10a, b). Accordingly, H&E staining showed that LY3000328 treatment reduced the cross-sectional area of the scar tissue (Fig. 1n, Supplementary Fig. S4h). IRF7-positive or CTSS-positive fibroblasts were detected in the mice hypertrophic scar (Supplementary Fig. S10c). IHC staining of the myofibroblast marker, α-SMA, as well as collagen I, collagen III, and fibronectin suggested that LY3000328 effectively inhibited myofibroblast activation (Fig. 1n, Supplementary Fig. S4i, Fig. S10d, e), which was suggestive of reduced scar formation.

We next examined the factors capable of up-regulating CTSS. RT-qPCR, WB, and IHC assays indicated an upregulated expression of interferon regulatory factor 7 (IRF7), a transcription factor among the 13 DEGs of the E18 group involved in the TLR signaling pathway (Fig. 1o, Supplementary Fig. S5a–h). The binding site of IRF7 to the CTSS promoter region was predicted (Fig. 1p). Luciferase activity in HSFs cells co-transfected with the CTSS promoter-WT and IRF7 was significantly increased, as compared to HSFs cells transfected with the CTSS promoter-WT and pcDNA. However, this capacity was lost in the mutant fragment of the same CTSS promoter (Fig. 1q). Results from ChIP analysis confirmed the endogenous binding of IRF7 to the CTSS promoter in HSFs cells (Supplementary Fig. S5i). We next transfected HSFs cells with an IRF7 overexpression plasmid, the overexpression of IRF7 was verified at mRNA and protein levels (Supplementary Fig. S6a, c, d). As expected, this overexpression of IRF7 greatly enhanced CTSS mRNA and protein expression (Supplementary Fig. S6b, c, e), as well as the mRNA and protein expression of Col 1, Col 3, and Fn (Supplementary Fig. S7a–g). To further confirm the joint regulatory function of the IRF7 and CTSS in scar generation, we next performed a reverse experiment. Cells were either transfected with an empty plasmid (Group I), the IRF7 overexpression plasmid alone (Group II), or a co-transfection with an IRF7 overexpression plasmid and CTSS knockdown plasmid (Group III). In Group III, the effect of IRF7 on downstream scar-related genes was counteracted by the down-regulation of CTSS (Fig. 1r, Supplementary Fig. S7h–l).

As expected, both IRF7 and CTSS were significantly increased in keloid tissue samples from adult patients by RT- PCR (Supplementary Fig. S8a, b) and WB assays (Fig. 1s, t, Supplementary Fig. S8c, d). IHC staining revealed that substantial increases in IRF7 and CTSS were detected in the dermis of keloid tissue, the same site where a large quantity of extracellular matrix accumulated (Supplementary Fig. S8e–g). This finding confirmed our conjecture that overexpression of IRF7 and CTSS may be critical factors in the formation of keloids. We acknowledge that as keloid do not often result from excisional wounds which was studied in the mouse model. Study on human hypertrophic scar is necessary, albeit it poses difficulties in obtaining clinical samples.

As shown in a schematic diagram, (Supplementary Fig. S9) we demonstrated the transcriptional activation effects of IRF7 on CTSS in fibroblasts and their potential as a therapeutic target for alleviating fibrosis. Of course, their interaction with other cellular and molecular component during the wound repair process, such as inflammatory monocytes, remains an intriguing topic for further investigation.^5^

### Supplementary information

Supplementary Materials

## Data Availability

The data that support the findings of this study are available from the lead corresponding author upon reasonable request. The data of RNA-Seq was deposited in the BioProject database of NCBI with the accession PRJNA833594.
