# Supplementary material for: IRF7 and CTSS are pivotal for cutaneous wound healing and may serve as therapeutic targets
Source: Signal Transduct Target Ther. 2023 Aug 30;8:322. doi: 10.1038/s41392-023-01517-1 (PMC10465541; doi:10.1038/s41392-023-01517-1)
Supplement: Supplementary file 1 — Supplementary Materials [file 41392_2023_1517_MOESM1_ESM.docx]

Supplementary Materials

IRF7 and CTSS are pivotal for cutaneous wound healing and may serve as therapeutic targets

Jiali Yin^1,2,5^ , Dongxin Shi^1,2^, Yan Sun^1,2^, Peiyao Zhu^1,2^, Yiping Zhao^1,2^, Xuegang Xu^1,2^, Hongduo Chen^1,2^, Yan Wu^1,2^*, Zhengwei Yuan^3^*, Xing-Hua Gao^1,2,4^*

1 Department of Dermatology, The First Hospital of China Medical University, Shenyang, China.

2 Key Laboratory of Immunodermatology, Ministry of Education and NHC; National joint Engineering Research Center for Theranostics of Immunological Skin Diseases, Shenyang, China.

3 Key Laboratory of Health Ministry for Congenital Malformation, Shengjing Hospital, China Medical University, Shenyang, China.

4 College of Medicine and Biological Information Engineering, Northeastern University, NO. 3-11, Wenhua Road, Heping District, Shenyang, China.

5 Department of Otolaryngology, Head and Neck Surgery, Yantai Yuhuangding Hospital, Qingdao University, Yantai, China.

Correspondence to: Yan Wu: [jlwuyan@126.com](mailto:jlwuyan@126.com) ; Zhengwei Yuan: [yuanzw@hotmail.com](mailto:yuanzw@hotmail.com) ; Xinghua Gao: [gaobarry@hotmail.com](mailto:gaobarry@hotmail.com)

**This PDF file includes:**

Materials and Methods

Figures. S1 to S9

Tables S1 to S2

Materials and Methods

**Human Skin Samples**

Clinically and pathologically confirmed Keloid tissue and surrounding normal-appearing skin were collected from 12 patients who underwent surgical resection. The procedures were approved by the Ethics Committee of the First Hospital of China Medical University and written consent was obtained from every participant.

**Fetal Mice Surgery and Hypertrophic Scar Modelling**

Six-week-old C57B1/6J mice, purchased from the Animal Center of Shengjing Hospital of China Medical University (CMU), received humanitarian treatment and mated overnight for timed gestations. Pregnancy begins with the appearance of a vaginal plug the next day 0.5 (E0). On the designated days of pregnancy (E16 and E18), the intrauterine operation was performed under anesthesia as previously described [1]. A purse string was made by a 7-0 suture above the dorsum of the fetus. Through a 3mm incision in the centrum of the purse-string suture, a full-thickness excision wound about 1mm in diameter was made on the dorsal side of the fetus and marked with Indian ink, which has been used to mark the site of injury for its stable, non-toxic, non-coagulative, non-fading properties[2]. During the procedure, the open area was rinsed with warm (37 °C) saline, and a blunt-tip insulin needle was used to replenish fluid into the damaged uterus while the purse was closed. After closing the abdominal cavity, mice were put in a warm chamber until they regained consciousness. As previously described [3], the mice were again euthanized 24 hours and 48 hours after the operation respectively, and tissue around the wound as well as the control skin of the same fetus was collected for the follow-up experiment. All procedures were in accordance of the National Institutes of Health Guide for the Care and Use of Laboratory Animals and approved by the Shengjing Hospital of CMU (Approval No. 2016PS113K).

The model of the hypertrophic scar was produced as previously reported [4, 5]. Briefly, a 2cm long full-thickness incision was created and sutured on the dorsal skin of adult C57B1/6J mice. The stitches were removed on day 4 and tension was achieved with clips on both sides parallel to the wound up to day 14. For interventional study, twelve mice were randomly divided into two groups. once a day from day 4 to day 14, the mice from the treatment group were intradermally injected with 100ul (10mg/kg dissolved in 10% DMSO) CTSS inhibitor (LY3000328, MedChemExpress), and the control mice were injected with the solvent. The mice were sacrificed and scar samples were obtained for subsequent experiments.

**Histological and Immunohistochemical Staining**

Fresh tissue was fixed with 4% paraformaldehyde and paraffin-embedded. Sections of 5um thickness were used for various staining approaches, i.e., hematoxylin and eosin (H&E), immunohistochemical (IHC) and Picrosirius red (PSR). PSR stained sections were observed under a green laser confocal scanning microscopy (CLSM) (Zeiss, Germany), to identify and quantify the collagen deposition in the intercellular matrix (ECM) around the injury and normal skin [6, 7]. IHC staining was carried out as detailed [8], Primary rabbit anti-IRF7 (1:200, Abcam) and rabbit anti-CTSS (1:200, Abcam) antibodies were used to detect IRF7 and CTSS in mice and human skin samples, and software ImageJ was used to quantify the positive area of immunohistochemical staining.

**RNA Sequencing and Bioinformatic Analysis**

Total RNA was isolated from wounded and control samples (n=3 per group at 24h after injury) using a Rneasy mini kit (Qiagen, Germany). RNA concentration, as well as purity, were determined by an Agilent 2100 Bioanalyzer (Agilent RNA 6000 Nano Kit, USA). The iatric Hiseq 2500 platform was used for RNA-seq (Shanghai Biotechnology Corporation). The differentially expressed genes (DEGs) were selected using the following filter criteria: FDR ≤0.05 and fold-change ≥2 [9]. Bioinformatics analysis was performed on DEGs, determined by the rich factor and P value < 0.05, including Gene Ontology (GO), Kyoto Encyclopedia of Genes and Genomes (KEGG). GO analysis was performed to obtain the classification of DEGs in Biological Process, Molecular Function, and Cellular Component. The top 10 terms of the icatricia process and enriched pathways are also presented.

**Cell Culture and Plasmid Transfection**

Human skin fibroblasts cells (HSFs, From Kunming Cell Bank, Chinese Academy of Sciences) were maintained in Dulbecco’s modified Eagle’s medium (DMEM; Gibco BRL) mixed with 10% fetal bovine serum (Gibco), 100 U/ml of penicillin, 100 mg/ml streptomycin and 1% MEM nonessential amino acids (MEM NEAA, Gibco) in a humidified incubator at 37℃ with 5% CO_2_.

HSFs cells were seeded into 6-well plates (approximately 1 × 10^5^ cells per well) and cultured before transfection. IRF7 overexpression plasmid and control (IRF7 & pcDNA) and CTSS knockdown plasmid and control (ShRNA01-03 & ShNC) were synthesized by SyngenTech (China). HSFs were transfected with 1.8 µg plasmids in 2 mL of culture medium per well, using JetPRIME (Polyplus Transfection). The cells were cultured for 48h before harvesting. Cells were maintained within 20 passages in all the experiments.

**Quantitative real-time RT-PCR**

Total RNA isolated from skin samples and HSFs cells was reverse-transcribed into cDNA with a PrimeScript RT kit (TaKaRa). RT-qPCR was conduct in a 7500 Real-time PCR system (ABI) as follows: pre-denaturation at 95°C for 5 min; 45 cycles at 95°C for 15 s and at 60°C for 45 s by using an SYBR green master mix kit (TaKaRa). Primers for mice or human genes were designed and synthesized by Sangon Biotech (Sangon Biotech, Shanghai, China) (Supplementary Table1).The relative expression of target genes was quantified using the 2- ΔΔCt method and GAPDH as a control.

**Western Blotting**

Protein was extracted from fetal mice skin, keloid tissue from the patients and normal skin or HSFs cells using radioimmunoprecipitation (RIPA) buffer (Solarbio, China). In brief, protein (about 30ug) was resolved by 10% or 8% sodium dodecyl sulfate-polyacrylamide gel electro-phoresis (SDS-PAGE) and then electroblotted onto the polyvinylidene difluoride (PVDF) membranes. After being blocked in 5% non-fat milk, the separated proteins were incubated with primary antibodies against IRF7 (1:2000, Abcam), CTSS (1:1000, Abcam), Collagen I (1:1000, Abcam), Collagen III (1:1000, ProteinTech), Fibronectin (1:1000, ProteinTech) or GAPDH (1:5000, ProteinTech) overnight at 4°C. Then membranes were washed and incubated with a secondary antibody (1:5000, Invitrogen) at room temperature for 1.5 hours. Positive bands were visualized by using enhanced chemiluminescent (ECL) reagent (Millipore) and detected by an imaging system (C300, USA).

**Luciferase Reporter Assay**

The possible binding sites of transcription factor IRF7 to CTSS promoter region were predicted using JASPAR and hTFtarget websites, and wild type and mutant sequences were synthesized. HSFs were transfected with either wild-type or mutant plasmids along with IRF7 overexpression plasmid or control. Renilla luciferase plasmid was used as endogenous control. 48 hours after transfection, the cells from each well were harvested. The combination of IRF7 and CTSS is determined by the dual-luciferase activity using a reporter assay system (Promega).

**Chromatin Immunoprecipitation (ChIP) Assay**

ChIP assay was performed according to the instructions from SimpleChIP Enzymatic Chromatin IP Kit (Cell Signaling Technology; No. 9002). In brief, after incubation with 1% formaldehyde, the cross-linking proteins to DNA were enzyme digested into fragments. After purification, part of the fragmented chromatin was taken out as input control and rotated overnight at 4℃ along with the other sample added with IRF7 antibody for immunoprecipitation. IgG was chosen as a negative control. Using the immunoprecipitation complex, the CTSS promoter fragment which has been confirmed by luciferase reporter assay was amplified by RT-qPCR. The two primers sequence were I CTSS-promoter1-F1: 5-CGTGTAAAACAGACAAAAGTAGAGGCT-3, CTSS-promoter-R1: 5-CACTTCTTCCTTTTAGGGCTTGCT-3, CTSS-promoter-F2: 5-ACAGACAAAAGTAGAGGCTAGAGTTC-3, CTSS-promoter1-R2: 5-CTTCTTCCTTTTAGGGCTTGCTTTT-3.

**Statistical Analysis**

Data were calculated as the mean ± SD of at least 3 independent repeats. Statistical analysis was carried out through SPSS 17.0 software (SPSS Inc., Chicago, IL, United States). Parametric data was compared by two-sample t-tests or one-way analysis of variance (ANOVA). p< 0.05 was considered as statistical significance.

**Data availability STATEMENT**

The authors declare that all data supporting the findings of this study are available within the article and its supplementary information files or from the corresponding author on reasonable request.

**References**

1.	Walmsley, G.G., et al., *A mouse fetal skin model of scarless wound repair.* J Vis Exp, 2015(95): p. 52297.

2.	Colwell, A.S., et al., *An in vivo mouse excisional wound model of scarless healing.* Plast Reconstr Surg, 2006. **117**(7): p. 2292-6.

3.	Hu, M.S., et al., *Pathway Analysis of Gene Expression in Murine Fetal and Adult Wounds.* Adv Wound Care (New Rochelle), 2018. **7**(8): p. 262-275.

4.	Shao, T., et al., *Research on function and mechanisms of a novel small molecule WG449E for hypertrophic scar.* J Eur Acad Dermatol Venereol, 2020. **34**(3): p. 608-618.

5.	Liu, X.J., et al., *Xiamenmycin attenuates hypertrophic scars by suppressing local inflammation and the effects of mechanical stress.* J Invest Dermatol, 2013. **133**(5): p. 1351-60.

6.	Khorasani, H., et al., *A quantitative approach to scar analysis.* Am J Pathol, 2011. **178**(2): p. 621-8.

7.	Zhang, D., et al., *Elastic, Persistently Moisture-Retentive, and Wearable Biomimetic Film Inspired by Fetal Scarless Repair for Promoting Skin Wound Healing.* ACS Appl Mater Interfaces, 2020. **12**(5): p. 5542-5556.

8.	Liu, Y.S., et al., *miR-322 treatment rescues cell apoptosis and neural tube defect formation through silencing NADPH oxidase 4.* CNS Neurosci Ther, 2020. **26**(9): p. 902-912.

9.	Fernando, R.L., et al., *Controlling the proportion of false positives in multiple dependent tests.* Genetics, 2004. **166**(1): p. 611-9.

**Figure.S1**

Fig. S1 **a-i.** The surgical procedure of intrauterine skin full-thick resection in fetal mice. **a-c.** Preparation and depilation of mouse abdomen. **d.** Exposure of uterus by midline laparotomy. **e.** Creation of purse string suture. **f.** Incision through uterine wall and 1 mm full-thickness excisional wound generation. **g.** Marking with India ink staining. **h.** Closure of purse string suture. **i.** Closure of abdomen. **j.** HE staining showing histological and inflammatory cell infiltration of wounds in the E16 and E18 mice 48 h post-injury (arrow indicates wound location as marked by Indian ink). k, l. Fractal dimension (FD) and *lacunarity* (L) analyses (n=6). The data were represented as means ± SDs. **P* < 0.05, ***P*< 0.01, scale bars: 20um.

**Figure. S2.**

Fig. S2 **a, b.** Top 10 enriched BP terms in GO analysis and pathways in KEGG analysis of the E16 group. **c.** Venn diagram of DEGs associated with the TLR signaling pathway in the E16 and E18 mice versus that of the total gene set. **d.** qRT-PCR results demonstrating the differential expression of genes in the TLR pathway in the E18 wounded region according to the transcriptome sequencing results, and the differential profile is consistent with the sequencing results (n=6). Data were represented as mean ± SD. **P* < 0.05, ***P* < 0.01.

**Figure. S3.**

Fig. S3 **a.** Quantification of Western blot bands of CTSS of E18 control and wound skin tissue (n=6). **b.** Quantification of CTSS protein in the E18 control and wound skin tissue. **c, d.** Western blot and quantitation of CTSS of E16 mice in the control and wounds skin tissue (n = 6). **e, f.** Representative immunohistochemical staining and quantitation for CTSS protein in E16 wounds and control. The red dotted line represents the defective skin tissue. Data were represented as mean ± SD. Scale bars: 20um. **P* < 0.05, ***P*<0.01.

**Figure. S4.**

Fig. S4 **a-c.** Effect of the CTSS knockdown plasmid, ShRNA01-03, on mRNA (**a**, n=3) and protein expression levels (**b**, **c**, n=3) in HSFs. **d-f.** Quantification of Collagen I (Col1), Collagen III (Col3) and Fibronectin (Fn) proteins in HSFs cells transfected with ShRNA or Control (n=3)**.** **g. Gross** measurement of scars (n=6). h,i. Quantification of scars and α-SMA protein expression. Data are shown as means ± SDs. **P* < 0.05, ***P*<0.01, *****P* < 0.0001.

**Figure. S5.**

Fig. S5 **a, b.** Western blot and quantification of IRF7 of E18 control and wound skin samples (n= 6). **c, d.** Representative immunohistochemical staining and quantification of IRF7 protein in E18 control and wound skin sections. Western blot and quantitation of IRF7 of E16 mice control and wounds skin tissue (n = 6). **e, f.** Western blot and quantitation of IRF7 of E16 mice control and wounds skin tissue (n = 6). **g, h.** Representative immunohistochemical staining and quantitation for IRF7 protein in E16 wounds and control. The red dotted line represents the defective skin tissue. **i.** Binding of IRF7 to CTSS promoters in HSFs as determined by the ChIP assay. Data represent mean ± SD. Scale bars: 20um. ***P*<0.01, ****P* < 0.001, *****P* < 0.0001.

**Figure. S6.**

Fig. S6 **a, b.** RT-qPCR analysis of mRNA level of IRF7 (**a**) and CTSS (**b**) in HSFs cells transfected with pcDNA or IRF7 (n=3). **c-e.** Western blot analysis (**c**) and quantification of IRF7 (**d**) and CTSS (**e**) protein levels in HSFs cells transfected with pcDNA or IRF7 (n=3). Data are shown as means ± SDs. **P* < 0.05, ***P*<0.01.

**Figure. S7.**

Fig. S7 **a-c.** RT-qPCR analysis of Col1, Col3 and Fn mRNA in HSFs cells transfected with pcDNA or IRF7 (n=3). **d**. Western blot detection of Col1, Col3 and Fn protein in HSFs cells transfected with pcDNA or IRF7. **e-g.** Quantification of Col1, Col3 and Fn (n=3). **h-l.** Quantification of IRF7, CTSS, Col1, COl3 and Fn protein (n=3). Data are shown as means ± SDs. **P* < 0.05, ***P*<0.01.

**Figure. S8.**

Fig. S8 **a, b.** Relative mRNA expression of IRF7 and CTSS in normal human skin and keloid tissue (n=12). **c, d.** Western blot quantification of IRF7 (**c**) and CTSS (**d**) in normal human skin and keloid tissue (n=8). **e.** Representative immunohistochemical staining for IRF7 and CTSS in normal human skin and keloid tissue. **f, g.** Quantification of IRF7 and CTSS protein expression levels (Scale bars: 20 um). Data are shown as means ± SDs. ***P*<0.01, ****P* < 0.001, *****P* < 0.0001.

**Figure. S9.**

Fig. S9 Schematic representation of the possible role of IRF7 and CTSS on scar formation. IRF7 acts as a key regulator in the transition from scarless to scarring in late embryonic stage. After skin injury, fibroblasts produce excess IRF7, which further activates the transcription and secretion of CTSS, contributing to the remodeling of the extracellular matrix, and leads to scarring. Intervention in CTSS expression can be effective in alleviating adult hypertrophic scars in mice. This schematic was drawn using Adobe Illustrator 2020.

**Figure. S10.**

Fig. S10 **a.** H&E staining of skin sections of the hypertrophic scar model at 40x magnification. Scale bar: 20µm; **b.** measurement of the thickness of the scar and the adjacent normal skin (n=6); **c.** Immunofluorescence detection for IRF7-positive or CTSS-positive fibroblasts in the mice hypertrophic scar. Arrows: IRF7 or CTSS with PDGFRA double staining-positive cells. PDGFRA, platelet derived growth factor receptor alpha. **d,e** Col1, Col3 and Fn IHC staining in normal control skin and hypertrophic scars (n=3). Scale bar: 20 µm. Data are shown as the means ± SDs. **P<0.01, ***P < 0.001.

**Supplementary Table 1. Primer sequences for RT-qPCR**

| **Genes** | **Species** | **Primer** | **Sequence** |
| --- | --- | --- | --- |
| *IRF7* | Mouse | Forward | 5′-AAATAGGGAAGAAGTGAGCCTC-3′ |
|  | Mouse | Reverse | 5′-CCCTTGTACATGATGGTCACAT-3′ |
| *CTSS* | Mouse | Forward | 5′-GAAGAAATCTTGTGTCGGATGG-3′ |
|  | Mouse | Reverse | 5′-CACAAGAACCCTGGTATTTCAC-3′ |
| *GAPDH* | Mouse | Forward | 5′-GGTTGTCTCCTGCGACTTCA-3′ |
|  | Mouse | Reverse | 5′-TGGTCCAGGGTTTCTTACTCC-3′ |
| *IRF7* | Human | Forward | 5′-GGAGAGGGACAAGAAGGGCT-3′ |
|  | Human | Reverse | 5′-CTCTACTGCCCACCCGTACA-3′ |
| *CTSS* | Human | Forward | 5′-GTGTGCTCTTGGTGTGCTCCTC-3′ |
|  | Human | Reverse | 5′-CTTCCAGAGATGCCAGTGGTGATC-3′ |
| *Collagen I* | Human | Forward | 5′-AAAGATGGACTCAACGGTCTC-3′ |
|  | Human | Reverse | 5′-CATCGTGAGCCTTCTCTTGAG-3′ |
| *Collagen III* | Human | Forward | 5′-CTCAGGGTGTCAAGGGTGAAAGTG-3′ |
|  | Human | Reverse | 5′-TGTACCAGCCAGACCAGGAAGAC-3′ |
| *Fibronectin* | Human | Forward | 5′-AATAGATGCAACGATCAGGACA-3′ |
|  | Human | Reverse | 5′-GCAGGTTTCCTCGATTATCCTT-3′ |
| *GAPDH* | Human | Forward | 5′-CAGGAGGCATTGCTGATGAT-3′ |
|  | Human | Reverse | 5′-GAAGGCTGGGGCTCATTT-3′ |

**Supplementary Table 2. Significant fold changes of DEGs in the TLR pathway of E18 skin samples**

| Gene Name | P value | Q value | log2FC | Up/down |
| --- | --- | --- | --- | --- |
| SPP1 | 7.39E-75 | 8.48E-71 | 7.121751442 | UP |
| CTSS | 7.55E-26 | 1.02E-22 | 2.358227707 | UP |
| TLR13 | 3.77E-19 | 2.70E-16 | 2.367916807 | UP |
| IL6 | 2.19E-09 | 4.94E-07 | 1.267011187 | UP |
| CD14  Pik3ap1  IL1b  TLR 1  CXCL10  TLR8  TLR9  TLR4  IRF7 | 3.22E-08  3.71E-07  4.62E-06  1.08E-05  1.20E-05  1.20E-05  8.54E-05  0.0001053  0.0002257 | 5.86E-06  5.50E-05  0.0005230  0.0010999  0.0012013  0.0012013  0.0065558  0.0079510  0.0157497 | 3.317702225  1.472629599  4.787308641  2.226422902  3.077204223  1.4779221  1.824901627  1.067520444  1.511937431 | UP  UP  UP  UP  UP  UP  UP  UP  UP |

**Supplementary Table 3. SIgnificant fold changes of DEGs of E16 skin samples in the TLR pathway**

| Gene Name | P value | Q value | log2FC | Up/down |
| --- | --- | --- | --- | --- |
| SPP1 | 1.71E-12 | 1.32E-08 | 5.888543219 | UP |
| IL1B | 0.000137145 | 0.047315176 | 4.481717496 | UP |
| CXCL10 | 0.000141342 | 0.048045898 | 3.376522297 | UP |
